# Supplementary material for: Towards inferring nanopore sequencing ionic currents from nucleotide chemical structures
Source: Nat Commun. 2021 Nov 11;12:6545. doi: 10.1038/s41467-021-26929-x (PMC8586022; doi:10.1038/s41467-021-26929-x)
Supplement: Supplementary file 1 — Supplementary Information [file 41467_2021_26929_MOESM1_ESM.pdf]

Towards Inferring Nanopore Sequencing Ionic Currents from Nucleotide Chemical Structures

Supplementary Information

Supplementary Figure 1. Goodness-of-fit of the canonical DNA analysis. Root Mean Square Error (RMSE) and Pearson correlation (r) values of DNA down-sample, base-dropout, position-dropout and model combination analyses. Run-1 (solid boxes) and Run-2 (dashed boxes) refer to two independent replicates. RMSE and r values for the predictions of all DNA 6mers (Overall), DNA 6mers in training set only (Train) and DNA 6mers in test set only (Test) were marked as black, red and blue, respectively. The median, minimum/maximum (excluding outliers) and first/third quartile values were shown by the boxplots. See METHODS for details.

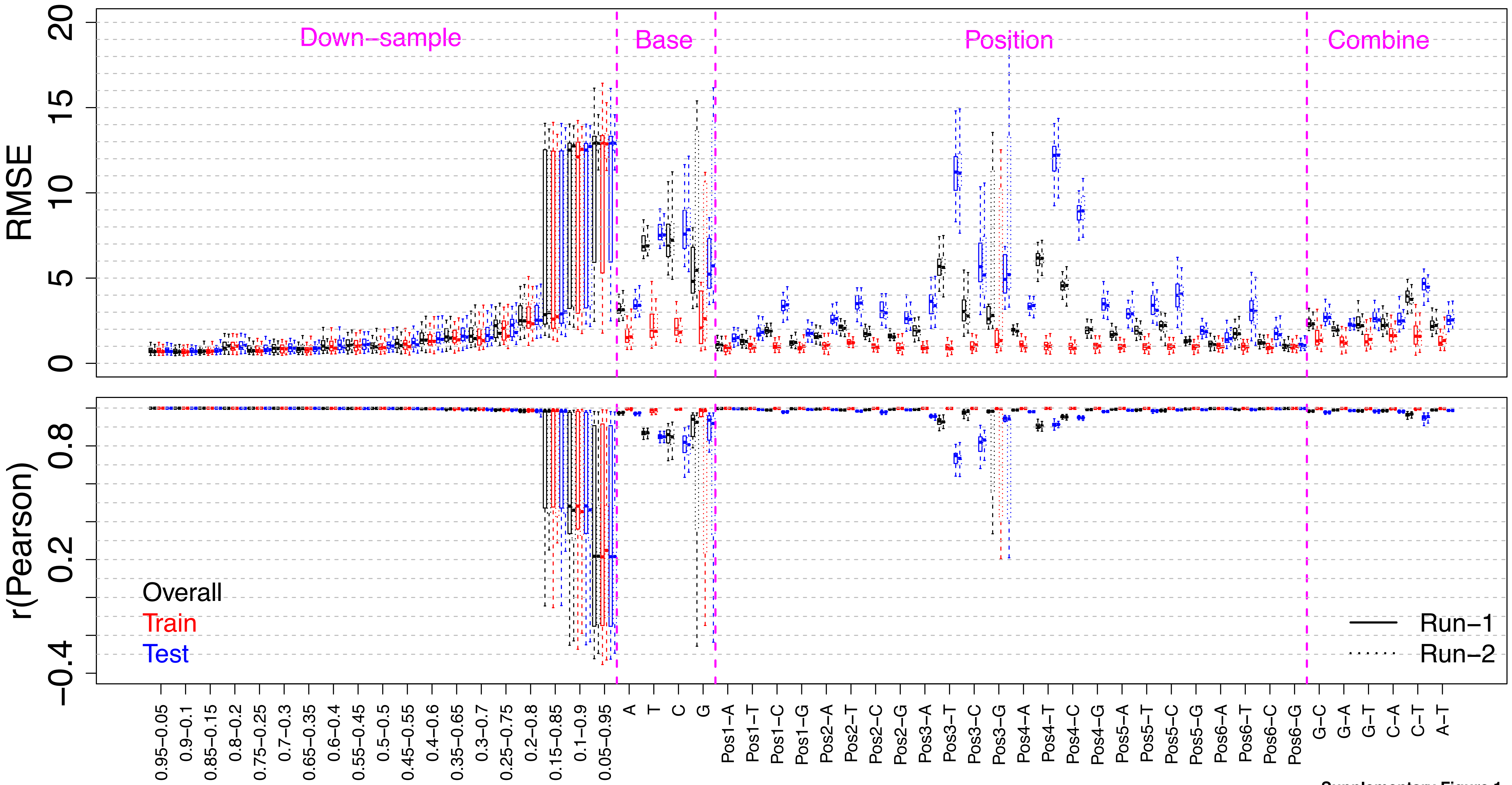

Supplementary Figure 1

Supplementary Figure 2. Goodness-of-fit of the canonical RNA analysis. Root Mean Square Error (RMSE) and Pearson correlation (r) values of DNA down-sample, base-dropout, position-dropout and model combination analyses. Run-1 (solid boxes) and Run-2 (dashed boxes) refer to two independent replicates. RMSE and r values for the predictions of all DNA 6mers (Overall), DNA 6mers in training set only (Train) and DNA 6mers in test set only (Test) were marked as black, red and blue, respectively. The median, minimum/maximum (excluding outliers) and first/third quartile values were shown by the boxplots. See METHODS for details.

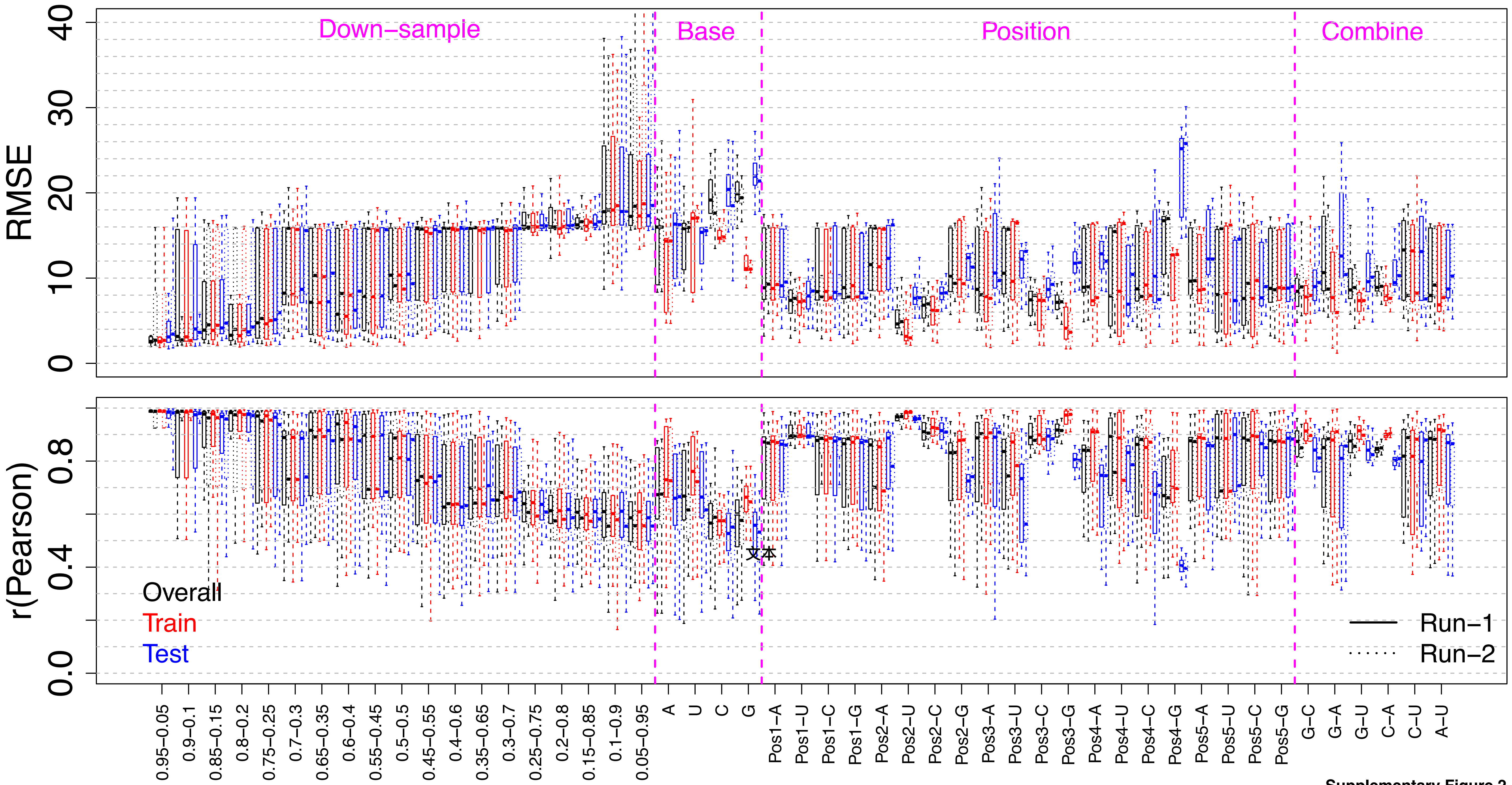

Supplementary Figure 2

Supplementary Figure 3. Goodness-of-fit of the DNA 5mC analysis. Root Mean Square Error (RMSE) and Pearson correlation (r) values of DNA 5mC-imputation analysis. These values were quantified against the nanopolish model [1,2]. Run-1 (solid boxes) and Run-2 (dashed boxes) refer to two independent replicates. RMSE and r values for the predictions of all DNA 6mers (Overall), DNA 6mers in training set only (Train) and DNA 6mers in test set only (Test) were marked as black, red and blue, respectively. The median, minimum/maximum (excluding outliers) and first/third quartile values were shown by the boxplots. See METHODS for details.

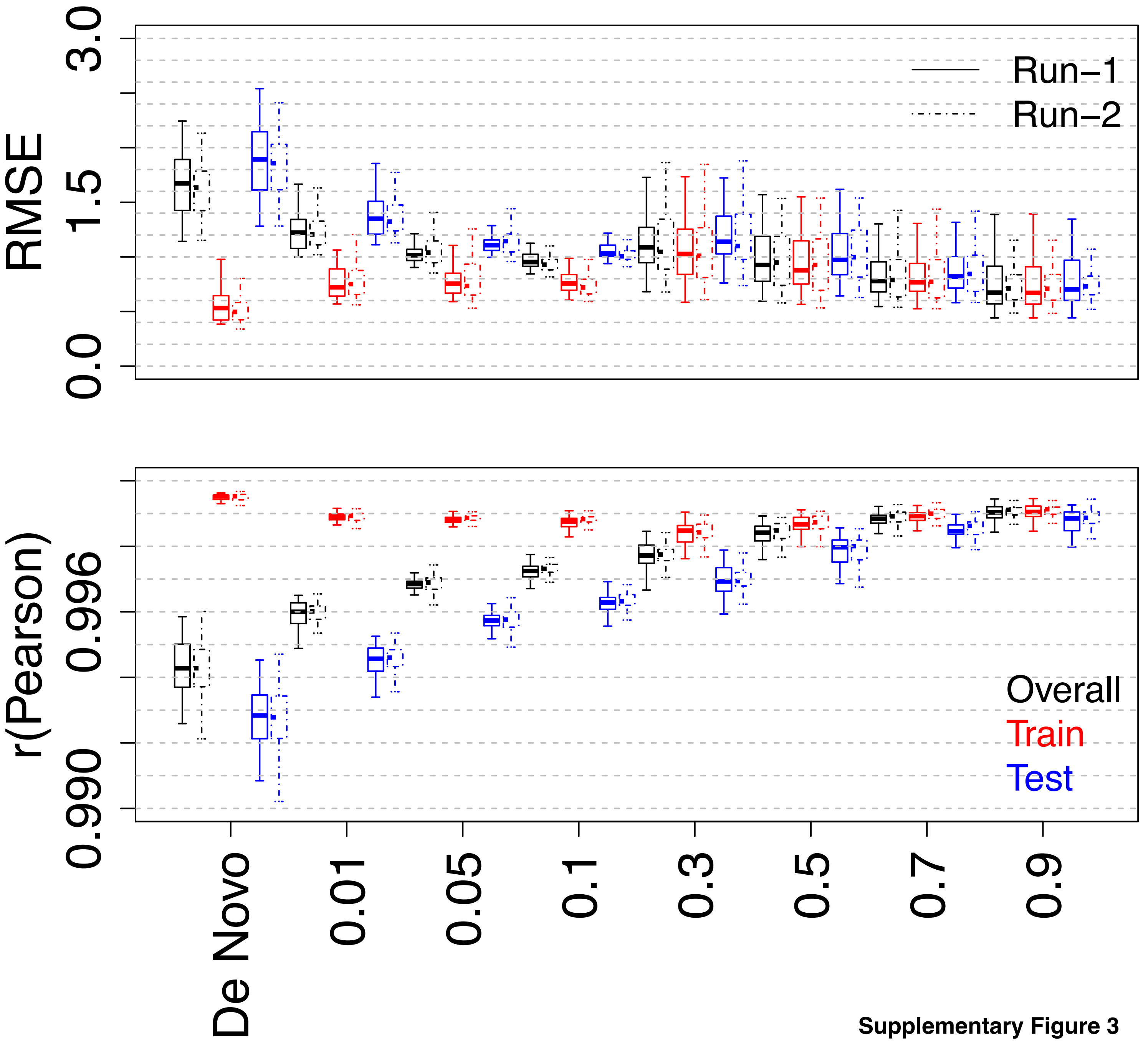

Supplementary Figure 3

Supplementary Figure 4. RMSE correlation in DNA 5mC-*de novo* analysis. For both Run-1 and Run-2, RMSE values obtained from canonical and 5mC-containing DNA 6mers were compared. Dots on the scatter-plots represent training-prediction repeats.

Supplementary Figure 4

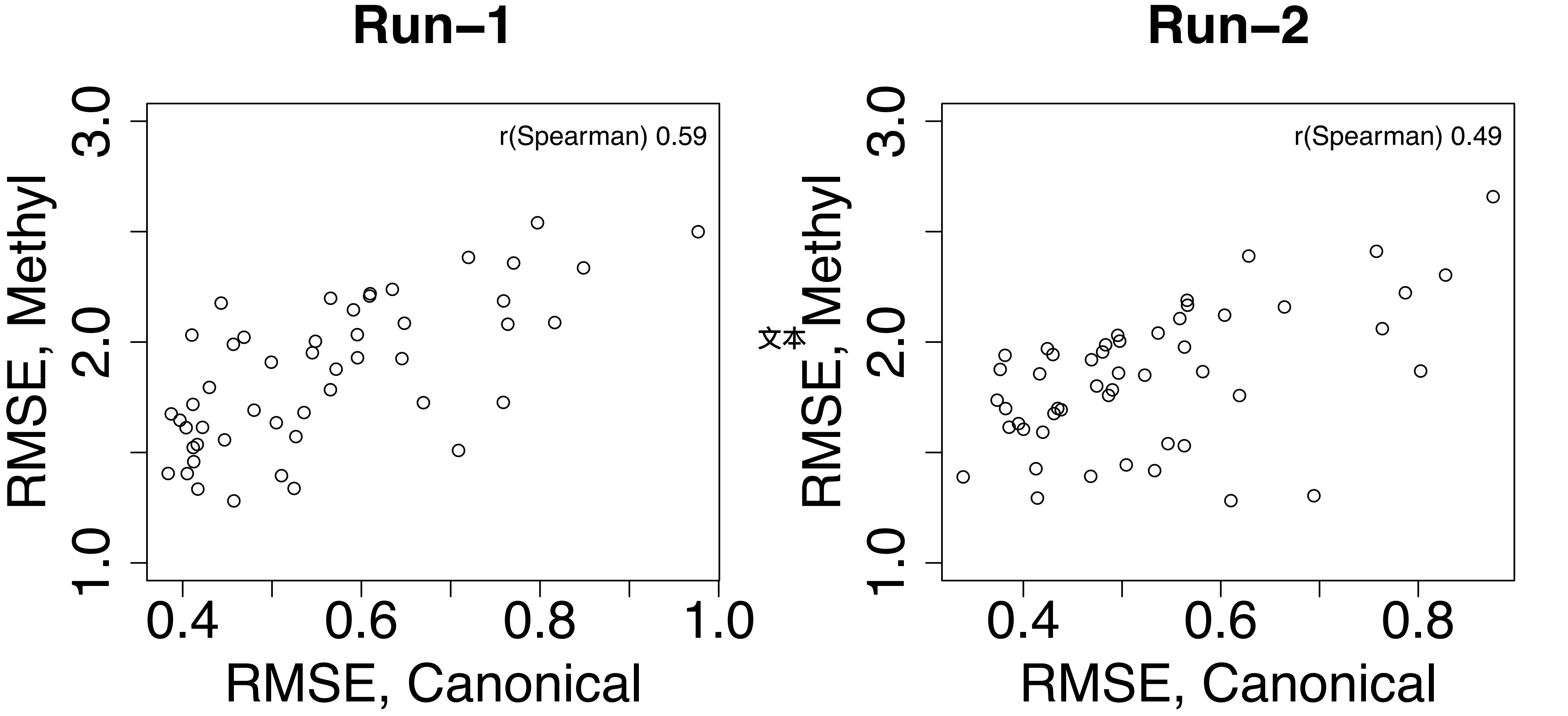

Supplementary Figure 5. Predictive accuracy of DNA 5mC analysis. Predictive accuracy was quantified by true positive rate (TPR), true negative rate (TNR), positive predictive value (PPV), negative predictive value (NPV), F1-score (F1) and balanced accuracy (BA). FAB39088 (black) and FAF01169 (red) refer to two independent NA12878 cell line native genomic DNA nanopore sequencing datasets [3]. Nanopolish refers to predictive analysis using the nanopolish model [1,2]. Megalodon refers to predictive analysis performed using the deep learning-based megalodon algorithm (https://github.com/nanoporetech/megalodon). The median, minimum/maximum (excluding outliers) and first/third quartile values were shown by the boxplots. See METHODS for details.

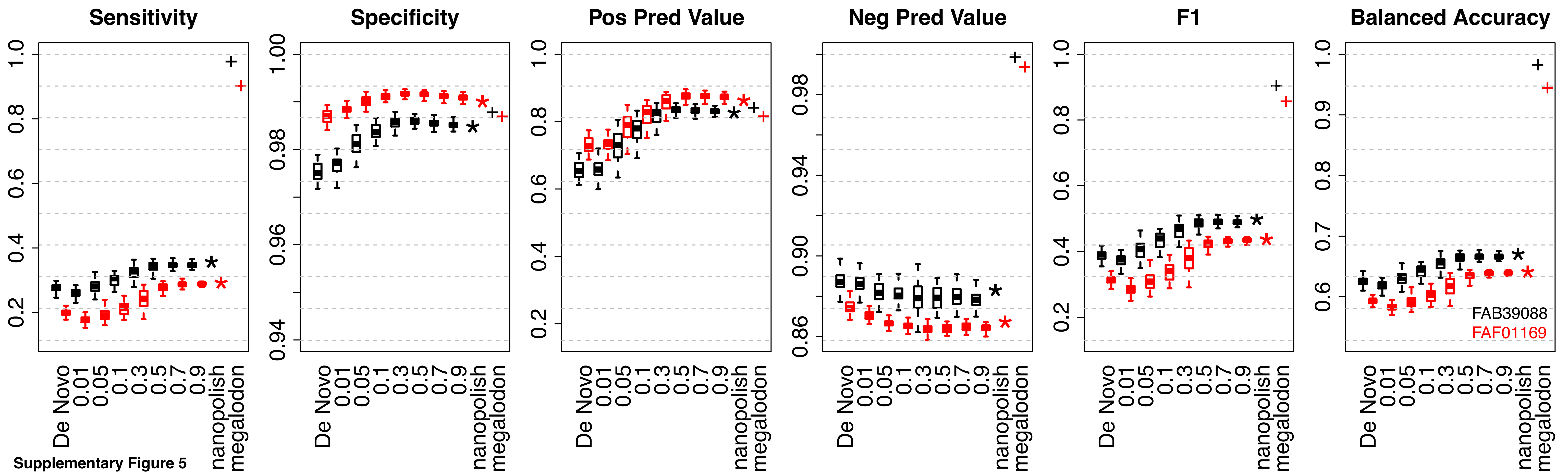

Supplementary Figure 5

Supplementary Figure 6. Visualizing canonical DNA 6mer atom similarity matrices. Without losing generality, we visualized the atom similarity matrices of 10 random canonical DNA 6mers. Similarity matrices were calculated using the Pearson correlation of the state vectors outputted by the final GCN layers. Corresponding chemical structures of analyzed DNA 6mers were shown side-by-side of the similarity matrices, based on which atoms were numbered and colored. Carbon, nitrogen, oxygen and phosphorus were colored as black, blue, red and orange, respectively.

Supplementary Figure 6

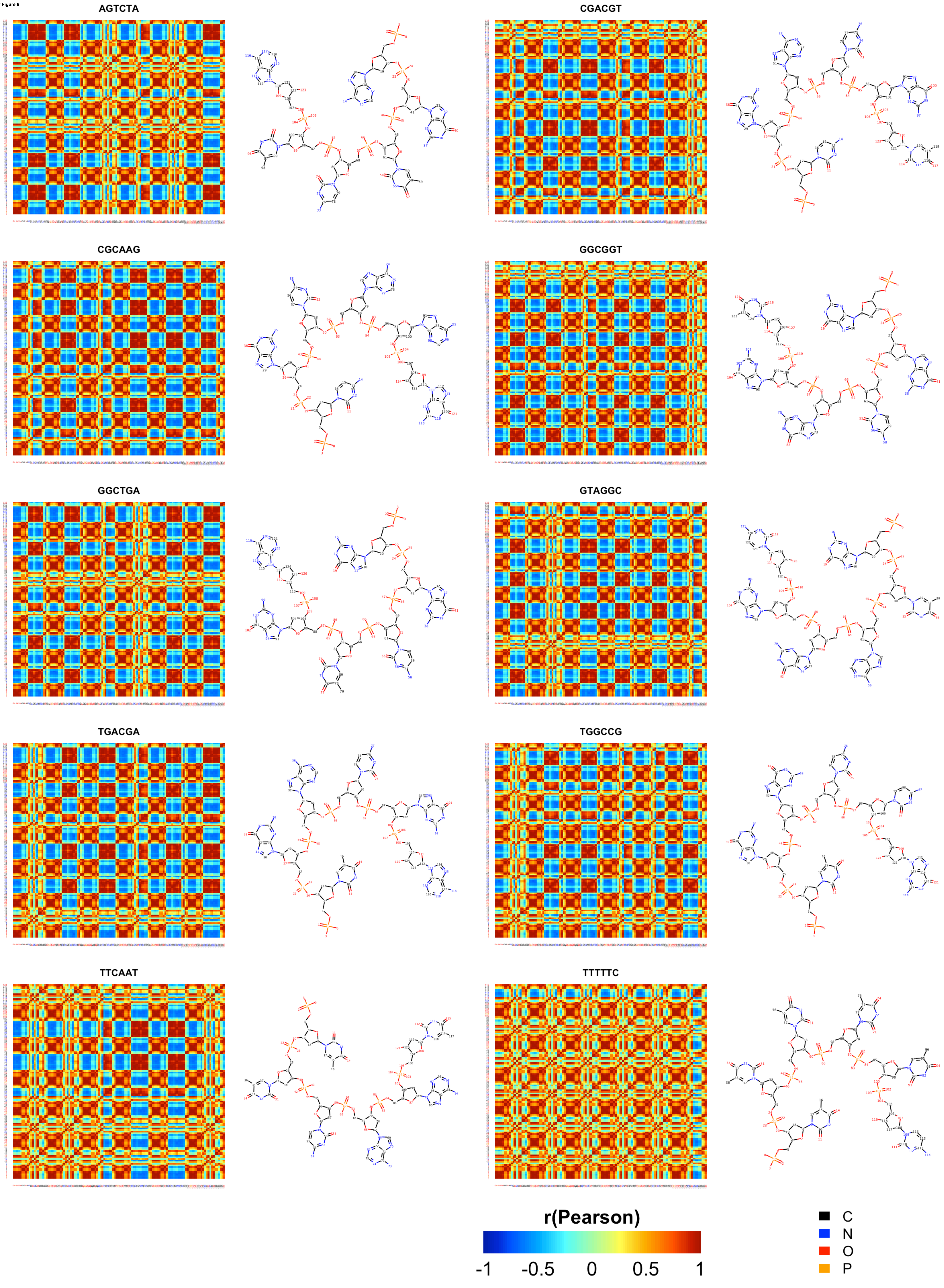

Supplementary Figure 7. Visualizing 5mC-containing DNA 6mer atom similarity matrices. Without losing generality, we visualized the atom similarity matrices of 10 random 5mC-containing DNA 6mers. 5mC was abbreviated as M for simplicity. Similarity matrices were calculated using the Pearson correlation of the state vectors outputted by the final GCN layers. Corresponding chemical structures of analyzed DNA 6mers were shown side-by-side of the similarity matrices, based on which atoms were numbered and colored. Carbon, nitrogen, oxygen and phosphorus were colored as black, blue, red and orange, respectively.

Supplementary Figure 7

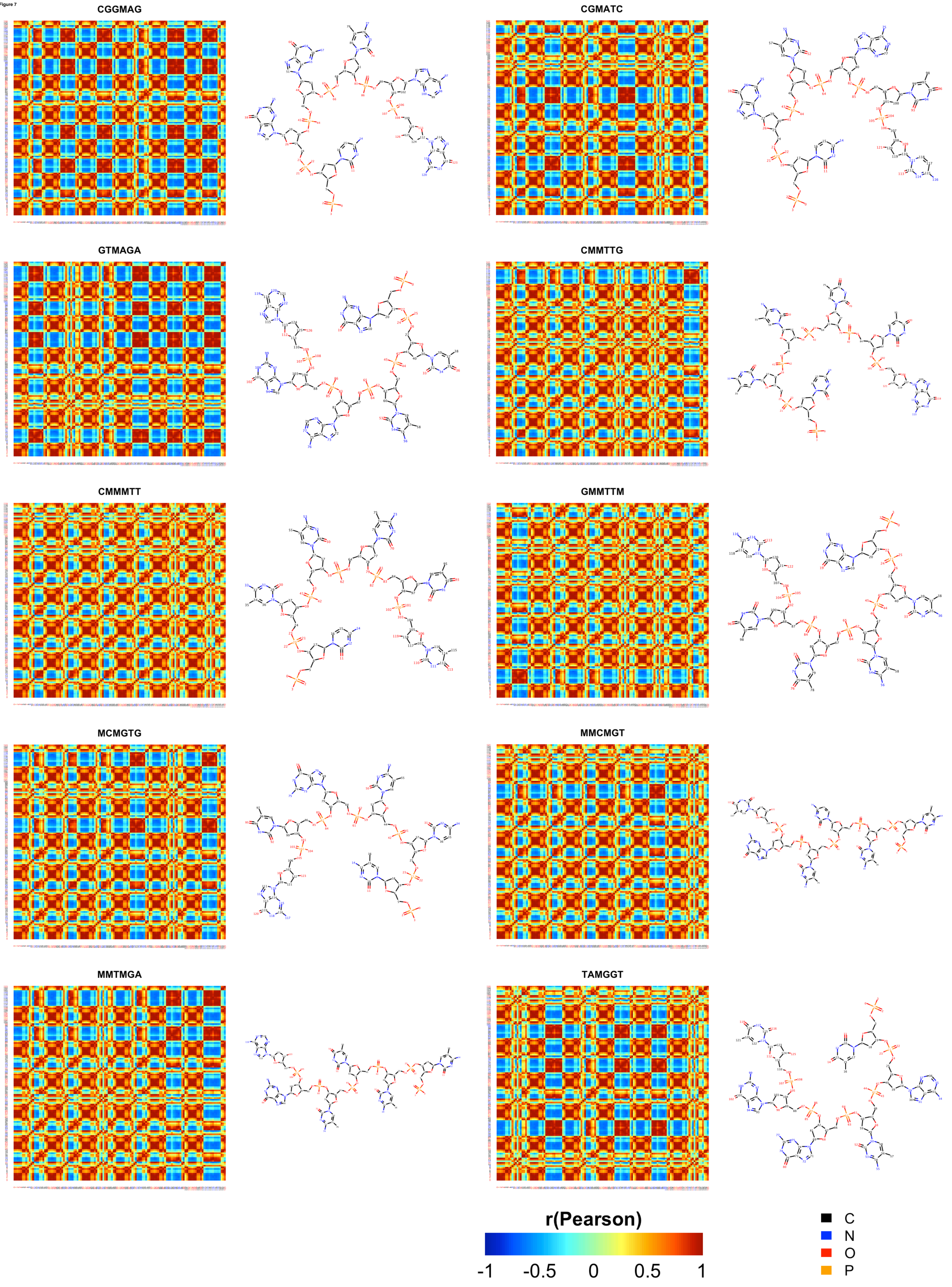

Supplementary Figure 8. Visualizing inter-kmer atom similarity matrices. Without losing generality, we analyzed the inter-kmer atom similarity between modified DNA 6mer GT(5mC)AGA and corresponding canonical counterpart GTCAGA. (A) Visualizing the inter-kmer similarity matrix, which was calculated using the Pearson correlation of the state vectors outputted by the final GCN layers. (B) The chemical structure of DNA 6mer GT(5mC)AGA. (C) The chemical structure of DNA 6mer GTCAGA. Based on chemical structures in (B) and (C) atoms were numbered and colored. Carbon, nitrogen, oxygen and phosphorus were colored as black, blue, red and orange, respectively.

Supplementary Figure 8

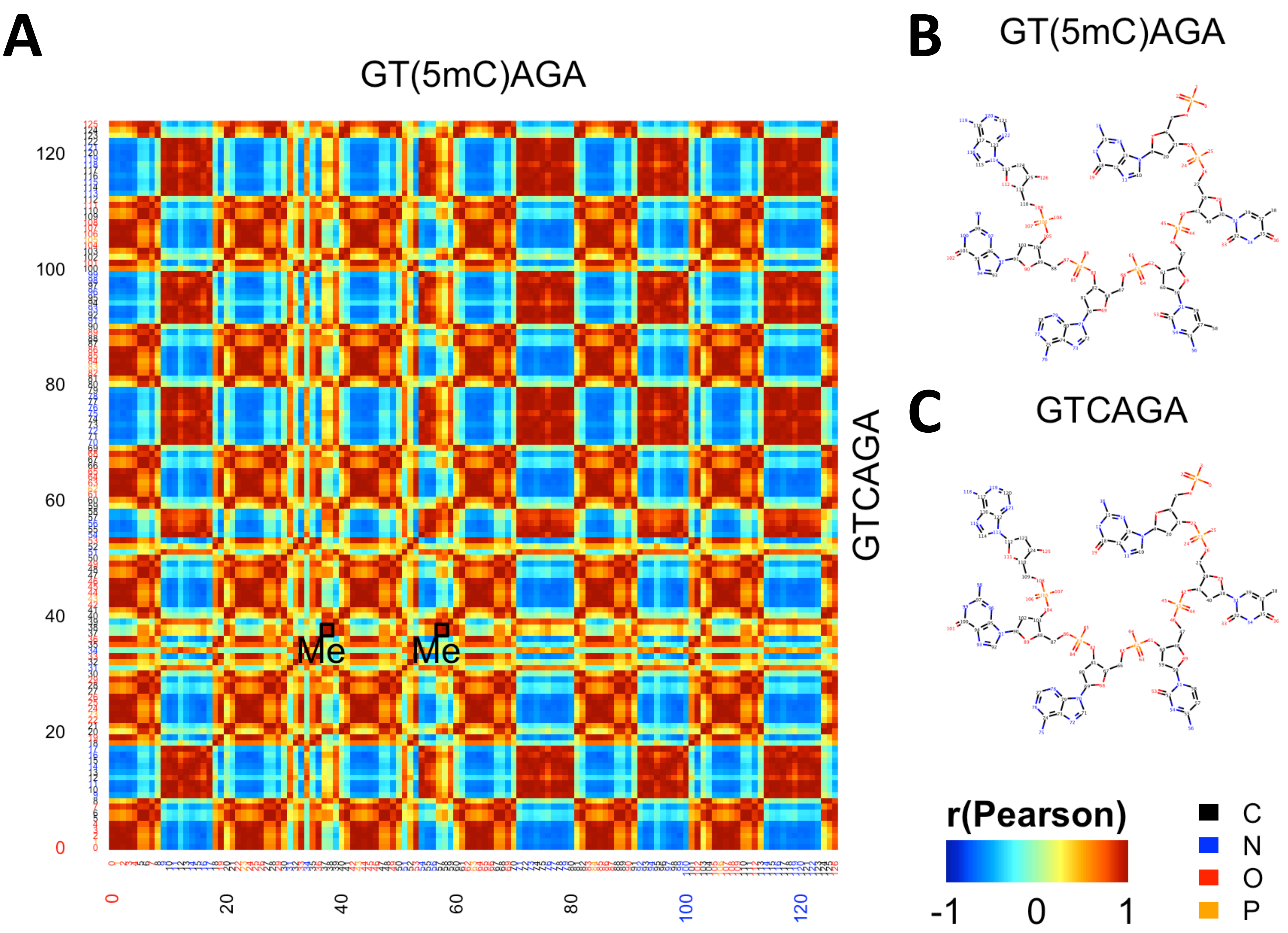

Supplementary Figure 9. RNA 2mG analysis. (A) The empirical ionic current signal distribution of RNA 5mer G(2mG)CCC, as well as the ONT ionic current signal distribution of pairing canonical RNA 5mer GCCCC were visualized in red and blue curves, respectively. Characteristic ionic current signals of G(2mG)CCC and GGCCC predicted by the deep learning framework were visualized in red and blue boxes, respectively. (B) For *E.coli* 16S rRNA transcript J01859.1 position 1206, 851, 1221, 1386, the fraction of modified (2mG) reads determined by signalAlign with predicted RNA 5mer ionic current signals was quantified. For boxplots in (A) and (B), the median, minimum/maximum (excluding outliers) and first/third quartile across the 50 prediction repeats were shown.

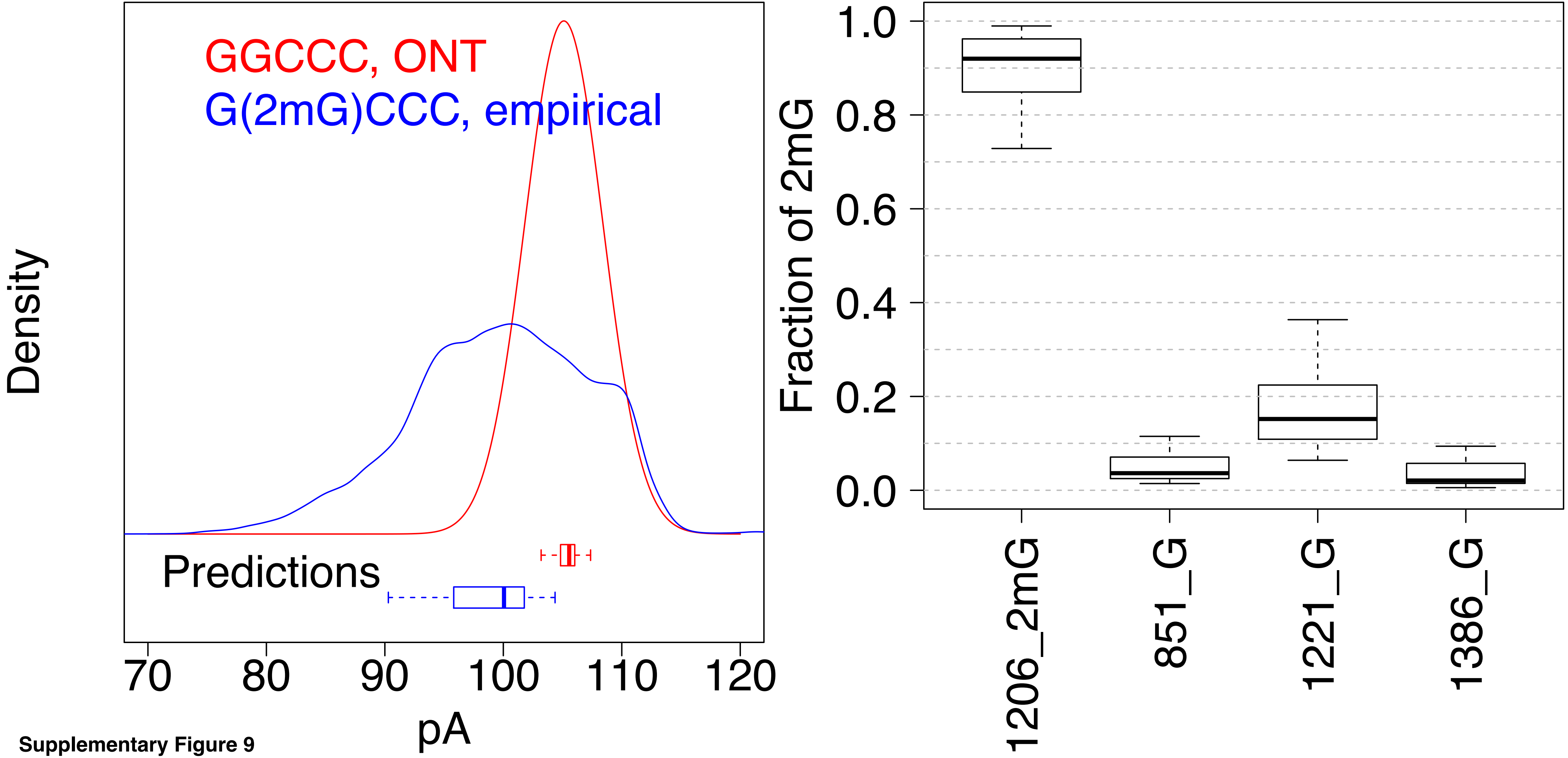

Supplementary Figure 9

Supplementary Figure 10. Chemical group stack analysis. Framework trained with all possible canonical DNA 6mers was used to predict 6mA-containing 6mers. 6mA-containing kmers were grouped by the positions of 6mAs. Signal distributions of 6mA-containing kmers and their canonical counterparts were shown in the boxplot. The median, minimum/maximum (excluding outliers) and first/third quartile values were shown by the boxplots. See METHODS for details.

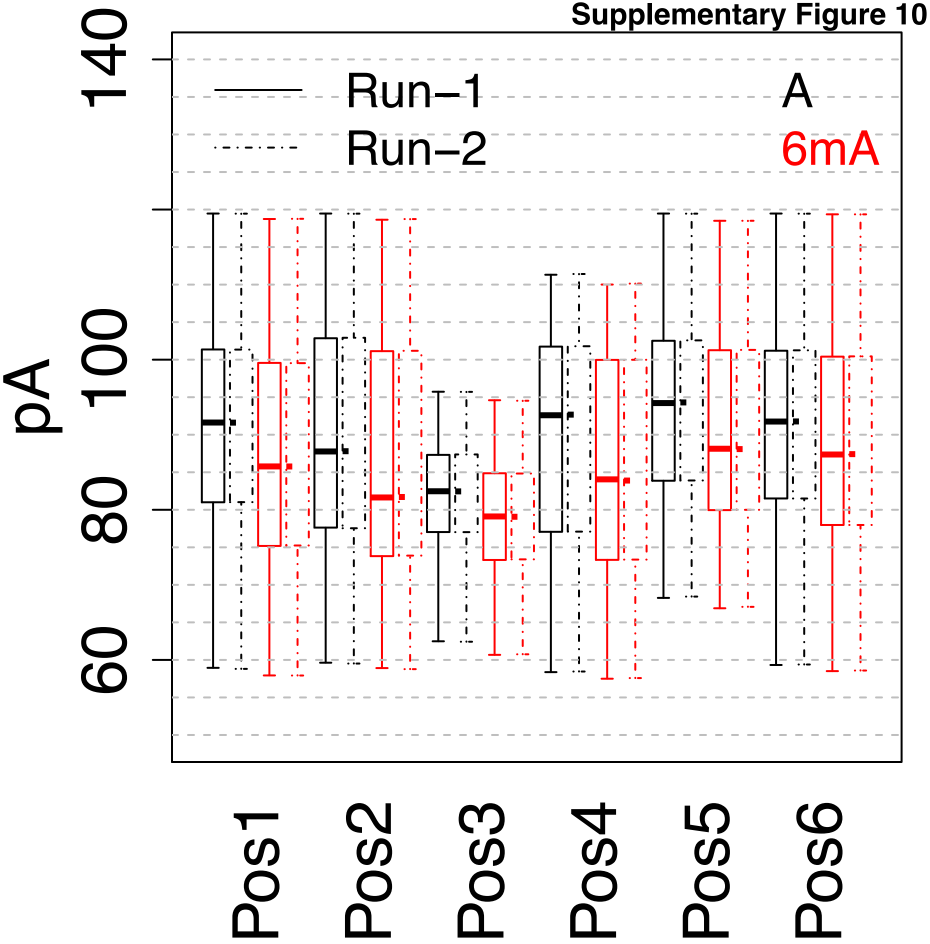
Run−1
Run−2
A
6mA
pA
60
80
100
140
Pos1
Pos2
Pos3
Pos4
Pos5
Pos6

Supplementary Table 1. Median RMSE and Pearson correlation values of the down-sample analysis.

	RMSE		r(Pearson)	
	Run–1	Run–2	Run–1	Run–2
0.05–0.95	12.93	12.92	0.2142	0.215
0.1–0.9	12.11	12.51	0.4823	0.4832
0.15–0.85	2.591	2.899	0.9874	0.9833
0.2–0.8	2.401	2.533	0.9875	0.9846
0.25–0.75	2.049	2.243	0.9918	0.9895
0.3–0.7	1.505	1.633	0.9964	0.9948
0.35–0.65	1.468	1.595	0.9959	0.9944
0.4–0.6	1.285	1.441	0.9968	0.9956
0.45–0.55	1.128	1.217	0.9979	0.9971
0.5–0.5	0.9553	1.05	0.9985	0.9978
0.55–0.45	0.9976	1.071	0.998	0.9974
0.6–0.4	0.9814	1.06	0.9983	0.9977
0.65–0.35	0.7809	0.8686	0.999	0.9985
0.7–0.3	0.8658	0.9145	0.9989	0.9985
0.75–0.25	0.7468	0.8009	0.9989	0.9987
0.8–0.2	0.9185	0.9629	0.9988	0.9984
0.85–0.15	0.6881	0.7183	0.9992	0.999
0.9–0.1	0.6765	0.7212	0.9991	0.9989
0.95–0.05	0.724	0.7601	0.9992	0.999

Supplementary Note 1. Goodness-of-fit of the canonical DNA analysis. We first evaluated whether the proposed framework could generalize information to nucleotides that were not present in the entire training data. We thus trained the framework using the DNA 6mers that do not contain each nucleotide (base-dropout, see METHODS). Such training sets retain ~18% of the total 6mers. Therefore we used the 0.2-0.8 train-test split as the baseline null model. As shown in Figure 1B and Supplementary Figure 1, base-dropouts significantly decreased the prediction power compared to the baseline null model. Such a result suggests that the four DNA nucleotides provide orthogonal information during training. In addition, the prediction power was more impaired by excluding T and C, which suggests that the four nucleotides have unequal importance.

We also evaluated the framework's generalizability to nucleotides that were not present in particular DNA 6mer positions (position-dropout, see METHODS). Such position-dropout retains 75% of total 6mers for training, so we used the 0.75-0.25 train-test split as the baseline null model. As shown in Figure 1B and Supplementary Figure 1, in general the prediction power was significantly impaired by excluding T and C, consistent with the nucleotide importance evaluated by base-dropout analysis. Meanwhile, dropouts in 3rd and 4th positions contributed the most to prediction power decrease, followed by 2nd and 5th positions. The positional importance suggested here was further consistent with [4].

We further explored whether full DNA 6mer models can be generalized by combining complementary base-dropout training sets, e.g. G-dropout and C-dropout that contains instances of C and G containing kmers, but no kmers containing both C and G (noted as G-C, see METHODS). Such training sets contain ~34% of total DNA 6mers, thus 0.35-0.65 train-test split was used as the baseline null model. As shown in Figure 1B and Supplementary Figure 1, in general the prediction power was comparable with the baseline null model, suggesting the validity of such model combination.

Supplementary Note 2. Goodness-of-fit of the canonical RNA analysis. Following the same pipeline as in DNA, down-sample, base-dropout, position-dropout and model combination analyses were also performed under RNA context. Meanwhile, RMSE and r were also used for prediction power evaluation for RNA analysis (see METHODS). Compared to DNA analysis, two major differences were observed. First, for RNA analysis as shown in Supplementary Figure 2, in general the prediction power was lower. For instance random down-sample analysis with 0.95-0.05 train-test split (best-performing random down-sample group), average RMSE values were ~0.8 and ~2.4 for DNA and RNA, respectively. We speculate that such prediction power difference was majorly caused by the number of training data points. As mentioned in

the main text, with the currently most prevalent Oxford Nanopore Technologies R9.4 nanopore sequencing chemistry, DNA is modeled with in total 4096 6mers. On the other hand, RNA is modeled with in total 1024 5mers, only 25% as opposed to the DNA scenario. Such fewer possible training data points might strongly compromise the prediction power of our framework. However, once trained with a similar amount of kmers, the RNA architecture could yield comparable prediction power. For instance, the RNA 0.95-0.05 (972 training kmers) and DNA 0.25-0.75 (1024 training kmers) train-test splits yielded comparable performance. Such a result suggested the validity of our proposed architecture.

The other major difference between DNA and RNA analysis is, the four canonical DNA bases (A, T, G, C) are "orthogonal" to each other (Figure 1B and Supplementary Figure 1). In contrast, base-dropout will not cause statistically significant decrease in prediction power, suggesting that the four canonical RNA bases (A, U, G, C) can complement each other in terms of their chemical properties (Supplementary Figure 2). Here "orthogonal" means base-dropouts will significantly decrease the prediction power as opposed to the corresponding random down-sample null model. Notably, such an "orthogonality effect" was particularly strong for T and C. We speculate that such a difference can be explained by the additional methyl group in T. Among the four DNA canonical bases, methyl only appears in T, thus cannot be compensated by other combining A, G and C. Similarly, considering such methyl is encoded with pyrimidine backbone (Figure 2A and Supplementary Figure 6), the representation of the other pyrimidine nucleobase, C is also affected. Thus T and C were more "orthogonal" compared to A and G. As for RNA, without the additional methyl, the four canonical RNA bases complement each other in terms of their chemical structures. Further, the chemical information generalization among bases guarantees the proper representation of RNA 5mers under base-dropout scenario, thus producing statistical insignificant prediction power as opposed to the corresponding null model.

Supplementary Note 3. Benchmarking human genome C/5mC-status predictive analysis with the megalodon algorithm. As described in Supplementary Figure 5, the recently released megalodon algorithm (https://github.com/nanoporetech/megalodon) could drastically increase the accuracy for NA12878 cell line C/5mC status prediction, therefore could be used as an additional ground truth for benchmarking our "human genome C/5mC-status predictive analysis". Compared to the ground truth established from bisulfite sequencing datasets [5], using megalodon predictions as ground truth has two prominent advantages: 1) the megalodon algorithm yields per-read per-site predictions, which could provide higher resolution as opposed to bisulfite sequencing ground truth when evaluating predictive accuracy. 2) The bisulfite sequencing ground truth was established from separate experiments thus potential biological/technical

batch effects could be the concerns. We therefore adopted the megalodon predictions as the additional per-read per-site C/5mC status ground truth. We yielded the following predictive accuracy, which is comparable with the result in Supplementary Figure 5.

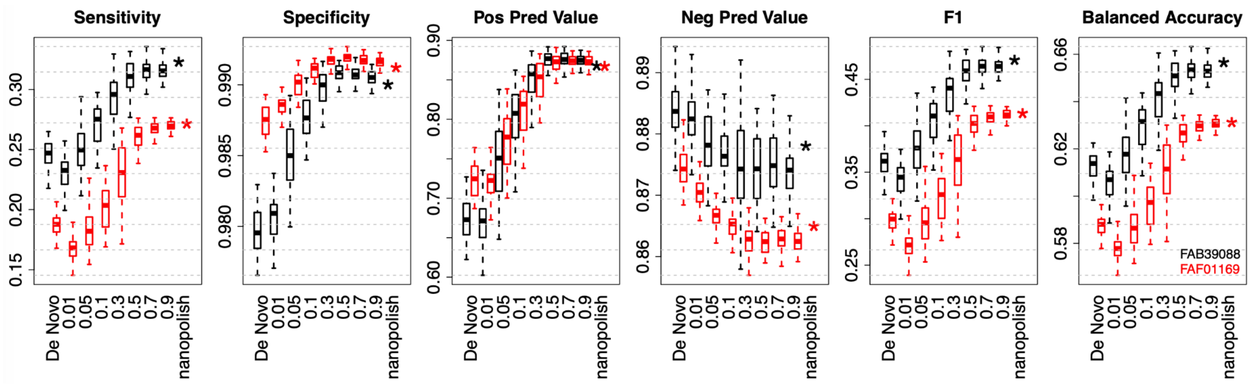

However, please note that the undermining limitation of the megalodon ground truth is the reliability of the megalodon predictions. Considering the algorithm has not been peer reviewed, as well as there are no available results benchmarking the performance under various scenarios, e.g. biological/technical replicates, different species, etc., we adopted the bisulfite sequencing ground truth throughout the study.

Supplementary Note 4. Building empirical kmer models. For nanopore sequencing chemistries after R9.4, the “official” ONT kmer models are no longer available. To solve such a problem, users could build empirical kmer models by the nanopore sequencing of synthesized control oligos.

The first question regarding building empirical kmer models is determining the effective kmer length (k). This could be done by following the procedures reported in our previous study [4].

The second question would be to determine the sequences of synthesized control oligos. Specifically, users need to decide 1) depth of nanopore sequencing, e.g. number of reads, and 2) kmers to be covered, e.g. the “minimal ideal kmer set”. These need to be done to 1) make sure full kmer models could be recapitulated from partial training using the deep learning architecture, and 2) save oligo synthesis and sequencing cost.

Please note that it’s crucially important that the control oligos cover all possible kmers. Otherwise, modification calling might be compromised. As shown in the following figure, we quantified the predictive accuracy of C/5mC status in a sequence context-specific manner. Specifically, with the same set of nanopore sequencing reads described in “human genome C/5mC-status predictive analysis” in METHODS, we quantified the balanced accuracy under **C**A, **C**T and **C**G contexts (**C**C motif is rare in human genome and was not covered by the selected reads). As shown in the following

figure, in general the predictive power of DNA kmer models inferred by our deep learning architecture (De Novo, 0.01, 0.05, 0.1, 0.3, 0.5, 0.7, 0.9) was comparable to the control nanopolish model. Meanwhile, kmer model-based signalAlign algorithm and deep learning-based megalodon algorithm yielded comparable predictive accuracy for **C**G context. However, signalAlign predictive accuracy was significantly compromised under **C**A and **C**T contexts. Moreover, predictive accuracy for **C**A and **C**T motifs was in general lower compared to the **C**G motif, even with the control nanopolish model. This is because the nanopolish model was constructed with the human genome, in which **C**A and **C**T motifs are less prevalent, and **C**C motif is rare. Therefore, **C**A, **C**T and **C**G contexts are less confidently represented in the nanopolish model.

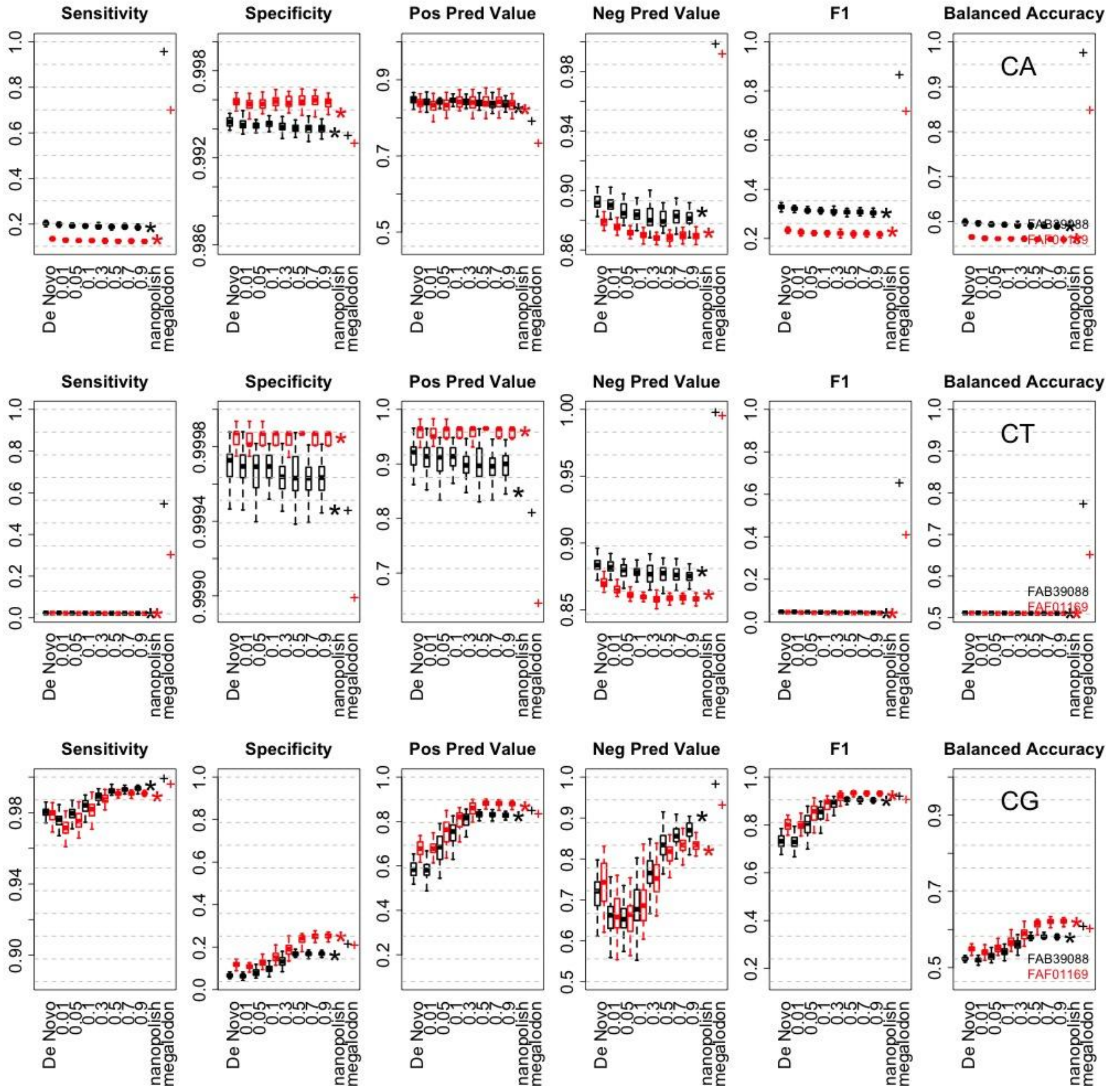

As for determining the proper depth of nanopore sequencing, we find it to be unnecessary: based on previous work, we believe that only modest sequencing is needed for building robust kmer models. Specifically, as reported in [6], the authors performed two parallel nanopore sequencing of synthesized RNA molecules, each with only a single MinION flowcell, further constructed corresponding {6mA, U, G, C} RNA 5mer models (two technical replicates). It was demonstrated that the two technical replicate RNA 5mer models are highly comparable, suggesting that robust kmer models could be generated from relatively small scale nanopore sequencing experiments.

As for finding the “minimal ideal kmer set”, although it might be a valuable idea to pursue, we find it to be less feasible. The reason being that the stochastic deep learning framework training process will introduce stochasticity in the prediction performance. That being said, the best prediction performance achieved by a certain training set could just be an effect of stochasticity, rather than the actual kmer composition. We also find the idea of finding the “minimal ideal kmer set” to be unnecessary, as kmers could be covered with relatively short sequences. As reported in the above-mentioned study [6], all possible {6mA, U, G, C} RNA 5mers could be covered with 4 sequences with average length ~2.5kb (2329, 2543, 2678, and 2795 bp, respectively).

Taken together, we believe that robust full kmer models could be built with affordable cost, following procedures reported in [6].

The last question would be to generate kmer models from nanopore sequencing readouts on the synthesized control oligos. We provide detailed procedures in the “kmer models” section of METHODS.

REFERENCES

1. Simpson, J. T., Workman, R. E., Zuzarte, P. C., David, M., Dursi, L. J., & Timp, W. (2017). Detecting DNA cytosine methylation using nanopore sequencing. Nature methods, 14(4), 407.
2. Loman, N. J., Quick, J., & Simpson, J. T. (2015). A complete bacterial genome assembled de novo using only nanopore sequencing data. Nature methods, 12(8), 733.
3. Jain, M., Koren, S., Miga, K. H., Quick, J., Rand, A. C., Sasani, T. A., ... & Malla, S. (2018). Nanopore sequencing and assembly of a human genome with ultra-long reads. Nature biotechnology, 36(4), 338.
4. Ding, H., Bailey, A. D., Jain, M., Olsen, H., & Paten, B. (2020). Gaussian Mixture Model-Based Unsupervised Nucleotide Modification Number Detection Using Nanopore Sequencing Readouts. Bioinformatics.
5. ENCODE Project Consortium. (2012). An integrated encyclopedia of DNA elements in the human genome. Nature, 489(7414), 57-74.

6. Liu, H., Begik, O., Lucas, M. C., Ramirez, J. M., Mason, C. E., Wiener, D., ... & Novoa, E. M. (2019). Accurate detection of m 6 A RNA modifications in native RNA sequences. Nature communications, 10(1), 1-9.
