# Supplementary material for: Towards inferring nanopore sequencing ionic currents from nucleotide chemical structures
Source: Nat Commun. 2021 Nov 11;12:6545. doi: 10.1038/s41467-021-26929-x (PMC8586022; doi:10.1038/s41467-021-26929-x)
Supplement: Supplementary file 3 — Reporting Summary [file 41467_2021_26929_MOESM3_ESM.pdf]

nature research

Corresponding author(s): Hongxu Ding
Joshua Stuart
Benedict Paten

Last updated by author(s): Sep 30, 2021

Reporting Summary

Nature Research wishes to improve the reproducibility of the work that we publish. This form provides structure for consistency and transparency in reporting. For further information on Nature Research policies, see our Editorial Policies and the Editorial Policy Checklist.

Statistics

For all statistical analyses, confirm that the following items are present in the figure legend, table legend, main text, or Methods section.

n/a	Confirmed	
[ ]	[x]	The exact sample size (*n*) for each experimental group/condition, given as a discrete number and unit of measurement
[x]	[ ]	A statement on whether measurements were taken from distinct samples or whether the same sample was measured repeatedly
[x]	[ ]	The statistical test(s) used AND whether they are one- or two-sided<br>*Only common tests should be described solely by name; describe more complex techniques in the Methods section.*
[x]	[ ]	A description of all covariates tested
[x]	[ ]	A description of any assumptions or corrections, such as tests of normality and adjustment for multiple comparisons
[ ]	[x]	A full description of the statistical parameters including central tendency (e.g. means) or other basic estimates (e.g. regression coefficient) AND variation (e.g. standard deviation) or associated estimates of uncertainty (e.g. confidence intervals)
[x]	[ ]	For null hypothesis testing, the test statistic (e.g. *F*, *t*, *r*) with confidence intervals, effect sizes, degrees of freedom and *P* value noted<br>*Give P values as exact values whenever suitable.*
[x]	[ ]	For Bayesian analysis, information on the choice of priors and Markov chain Monte Carlo settings
[x]	[ ]	For hierarchical and complex designs, identification of the appropriate level for tests and full reporting of outcomes
[ ]	[x]	Estimates of effect sizes (e.g. Cohen's *d*, Pearson's *r*), indicating how they were calculated

Our web collection on statistics for biologists contains articles on many of the points above.

Software and code

Policy information about availability of computer code

Data collection	No software was used for data collection.
Data analysis	Codes for constructing, training and running the deep learning framework are available at https://github.com/ioannisa92/Nanopore_modification_inference. Codes for nanopore sequencing data analysis are available at https://github.com/adbailey4/functional_model_analysis. Specifically, we adapted the original nanopolish (0.11.1) for our analysis. The adapted nanopolish is available at https://github.com/adbailey4/nanopolish. Codes for reproducing all figures are available upon request to the corresponding authors.<br><br>In addition, we used publicly available Keras (2.3.1), minimap2 (2.16-r922) and samtools (1.12) and nanopolish (0.11.1) in our analysis.

For manuscripts utilizing custom algorithms or software that are central to the research but not yet described in published literature, software must be made available to editors and reviewers. We strongly encourage code deposition in a community repository (e.g. GitHub). See the Nature Research guidelines for submitting code & software for further information.

Data

Policy information about availability of data

All manuscripts must include a data availability statement. This statement should provide the following information, where applicable:

- Accession codes, unique identifiers, or web links for publicly available datasets
- A list of figures that have associated raw data
- A description of any restrictions on data availability

The FAB39088 and FAF01169 NA12878 cell line native genomic DNA nanopore sequencing datasets were downloaded from https://github.com/nanopore-wgs-consortium/NA12878/blob/master/Genome.md. The two independent NA12878 bisulfite datasets were downloaded from https://www.encodeproject.org/experiments/ENCSR890UQO/. The E.coli 16S rRNA nanopore sequencing dataset was reported by [Smith et al. PloS one 2019]. The nanopore sequencing dataset

used to construct the GSE124309 model is available at GEO under the accession code GSE124309.

Field-specific reporting

Please select the one below that is the best fit for your research. If you are not sure, read the appropriate sections before making your selection.

[x] Life sciences [ ] Behavioural & social sciences [ ] Ecological, evolutionary & environmental sciences

For a reference copy of the document with all sections, see nature.com/documents/nr-reporting-summary-flat.pdf

Life sciences study design

All studies must disclose on these points even when the disclosure is negative.

Sample size	For every analysis, we trained 2x50 independent deep learning models. Since the performance difference between the two sets of 50 models is negligible, we thus consider n=50 sufficient.
Data exclusions	No data were excluded from the analyses.
Replication	For every analysis, we trained 2x50 independent deep learning models. Since the performance difference between the two sets of 50 models is negligible, we thus confirm the reproducibility.
Randomization	No participants involved in the study so randomization is not applicable.
Blinding	No participants involved in the study so blinding is not applicable.

Reporting for specific materials, systems and methods

We require information from authors about some types of materials, experimental systems and methods used in many studies. Here, indicate whether each material, system or method listed is relevant to your study. If you are not sure if a list item applies to your research, read the appropriate section before selecting a response.

Materials & experimental systems

n/a	Involved in the study	
[x]	[ ]	Antibodies
[x]	[ ]	Eukaryotic cell lines
[x]	[ ]	Palaeontology and archaeology
[x]	[ ]	Animals and other organisms
[x]	[ ]	Human research participants
[x]	[ ]	Clinical data
[x]	[ ]	Dual use research of concern

Methods

n/a	Involved in the study	
[x]	[ ]	ChIP-seq
[x]	[ ]	Flow cytometry
[x]	[ ]	MRI-based neuroimaging
